# First Satellite Tracking of a Juvenile Sandbar Shark (*Carcharhinus plumbeus*) off the Mediterranean Coast of Türkiye

**DOI:** 10.7717/peerj.21658

**Published:** 2026-08-18

**Authors:** Nuri Başusta, Loriane Mendez, Eva Jacquesson, Alen Soldo, Elizabeth Grace Tunka Bengil, Caner Enver Özyurt, Sinan Mavruk, Aytaç Özgül, Altan Lök, Eleonora de Sabata, Simona Clò

**Affiliations:** 1 Fisheries Basic Sciences, Faculty of Fisheries, Firat (Euphrates) University, Elazığ, Türkiye; 2 Commission Internationale pour l’Exploration Scientifique de la Mer Méditerranée (CIESM), Monaco, Monaco; 3 Centre Scientifique de Monaco, Monaco, Monaco; 4 Department of Marine Studies, University of Split, Split, Croatia; 5 Faculty of Marine Science, Ordu University, Ordu, Türkiye; 6 Faculty of Fisheries, Cukurova University, Adana, Türkiye; 7 Faculty of Fisheries, Ege University, Izmir, Türkiye; 8 MedSharks, Rome, Italy; 9 Stazione Zoologica Anton Dohrn, Napoli, Italy

**Keywords:** Satellite telemetry, Spatial behaviour, Elasmobranchs, *Carcharhinus plumbeus*, Sandbar shark, Iskenderun Bay, Levant Basin, Eastern Mediterranean

## Abstract

The spatial behavior of sharks in the Mediterranean Sea remains poorly understood, including for species listed as Endangered on the International Union for Conservation of Nature’s Red List, such as the sandbar shark (*Carcharhinus plumbeus*). To help address this knowledge gap, a field campaign was conducted in June 2024 in Yumurtalık Cove (Iskenderun Bay, eastern Mediterranean), one of the few confirmed breeding and nursery grounds for this species. During the mission, six sandbar sharks were captured using traditional longline methods. All individuals were measured onboard, and five were released due to their small size or compromised condition. A MiniPAT satellite tag (Wildlife Computers) was deployed on a juvenile, enabling analysis of horizontal and vertical movements over a 51-day tracking period. The shark traveled a maximum straight-line distance of approximately 88 km, moving toward Mersin Bay (Türkiye). The restricted movement pattern indicates that this young shark is more likely to exhibit resident or semi-resident behavior rather than undertaking long-distance migrations. Expanding long-term tracking efforts across multiple tagged individuals will be essential for clarifying habitat use and connectivity, ultimately supporting effective conservation of sandbar sharks in the Mediterranean Sea.

## Introduction

Elasmobranchs (sharks, skates and rays) are not only remarkable emblematic marine animals but also crucial indicators of ocean health, playing a key role in maintaining the balance and functioning of marine ecosystems. Their K-selected life-history strategy—characterized by slow growth, late maturity, and the production of few offspring with relatively high survival—greatly limits their ability to withstand pressures such as overfishing, climate change, habitat degradation and pollution, all of which contribute to population declines (García, Lucifora & Myers, 2008; Ferretti et al., 2010; Dulvy et al., 2014; Shea et al., 2024; Bengil & Başusta, 2024). In the Mediterranean Sea, sharks continue to be caught both intentionally and incidentally despite regional protections established under the Bern, Bonn, and Barcelona Conventions (Bengil & Başusta, 2024; Golani, Öztürk & Başusta, 2006; Rigby et al., 2021; Serena, Mancusi & Barone, 2024). At the national level, 27 shark and ray species are protected in Turkish waters under Fisheries Legislation No. 1380 (Official Notice for Regulating Commercial Fisheries of Water Resources, No. 6/1) (Başusta et al., 2024). Although targeted shark fisheries are rare in the Mediterranean and nonexistent for consumption in Türkiye, bycatch and discarding in coastal and industrial fisheries remain the primary threats to their populations (Bradai, Saidi & Enajjar, 2018; Carpentieri et al., 2021).

The sandbar shark (*Carcharhinus plumbeus* (Nardo, 1827)) is an active swimmer on sandy or muddy substrate that occasionally rises to the surface to feed on fishes, crustaceans and cephalopods (Saïdi et al., 2007). Classified as Endangered both in the Mediterranean and globally by the IUCN Red List of Threatened Species (Rigby et al., 2021), the sandbar shark has a worldwide tropical and temperate distribution except for the eastern Pacific (Golani, Öztürk & Başusta, 2006; Rigby et al., 2021). The coasts of Gabès Bay (southern Tunisia), Boncuk Cove (Gökova Bay, Türkiye) and Yumurtalık Cove (Iskenderun Bay, Türkiye) located in the central and eastern Mediterranean, have been confirmed as breeding and nursery areas for sandbar shark populations (Başusta, Başusta & Ozyurt, 2021). A major concern is the continued fishing pressure on these critical habitats, which threatens the survival and recovery of the species in the region (Saïdi et al., 2007; Başusta, Başusta & Ozyurt, 2021).

Cutting-edge tools such as satellite telemetry are increasingly used to investigate species’ spatial ecology, movement patterns, habitat use, and connectivity, while also providing valuable information to support conservation planning and assess the pressures affecting marine populations. This innovative science is helping to secure their future by improving monitoring and management strategies. Satellite tracking provides valuable opportunities to advance knowledge of species’ spatial ecology, thereby generating insights that can guide and strengthen conservation measures for vulnerable populations. Telemetry has become a widely used approach for studying the movement ecology, habitat use, and connectivity of aquatic animals, providing valuable information for fisheries management and conservation (Hussey et al., 2015; Queiroz et al., 2019; Özgül et al., 2024). These technologies have substantially improved our understanding of the spatial ecology of marine predators and their responses to environmental conditions and anthropogenic pressures, thereby supporting evidence-based monitoring and management strategies. Despite these advances, relatively few studies have focused on Mediterranean populations of sharks (Carbonara et al., 2024; Carbonara et al., 2026; Shea et al., 2024) or rays (Di Sciara et al., 2025).

Previous telemetry studies have shown that juvenile sandbar sharks exhibit high site fidelity and relatively restricted movements within coastal nursery habitats, whereas larger individuals may undertake broader seasonal movements (Rechisky & Wetherbee, 2003; Conrath & Musick, 2010). Such nursery areas provide critical habitats that support juvenile survival, growth, and recruitment, making their identification and protection a conservation priority (Heupel, Carlson & Simpfendorfer, 2007). Recent studies have further emphasized the importance of identifying and mapping nursery habitats for Mediterranean populations of the endangered sandbar shark to support effective conservation planning (Barbato et al., 2023). Nevertheless, telemetry information on Mediterranean populations of *Carcharhinus plumbeus* remains extremely limited, restricting our understanding of their movement ecology, habitat use, and connectivity. This study reports the locations and morphometric measurements of six sandbar sharks caught in Yumurtalık Cove, Iskenderun Bay, one of the few confirmed breeding and nursery grounds for the species in the Mediterranean Sea (Başusta, Başusta & Ozyurt, 2021) and presents the first satellite track of a tagged juvenile.

## Materials and Methods

### Study area

**Figure 1:**
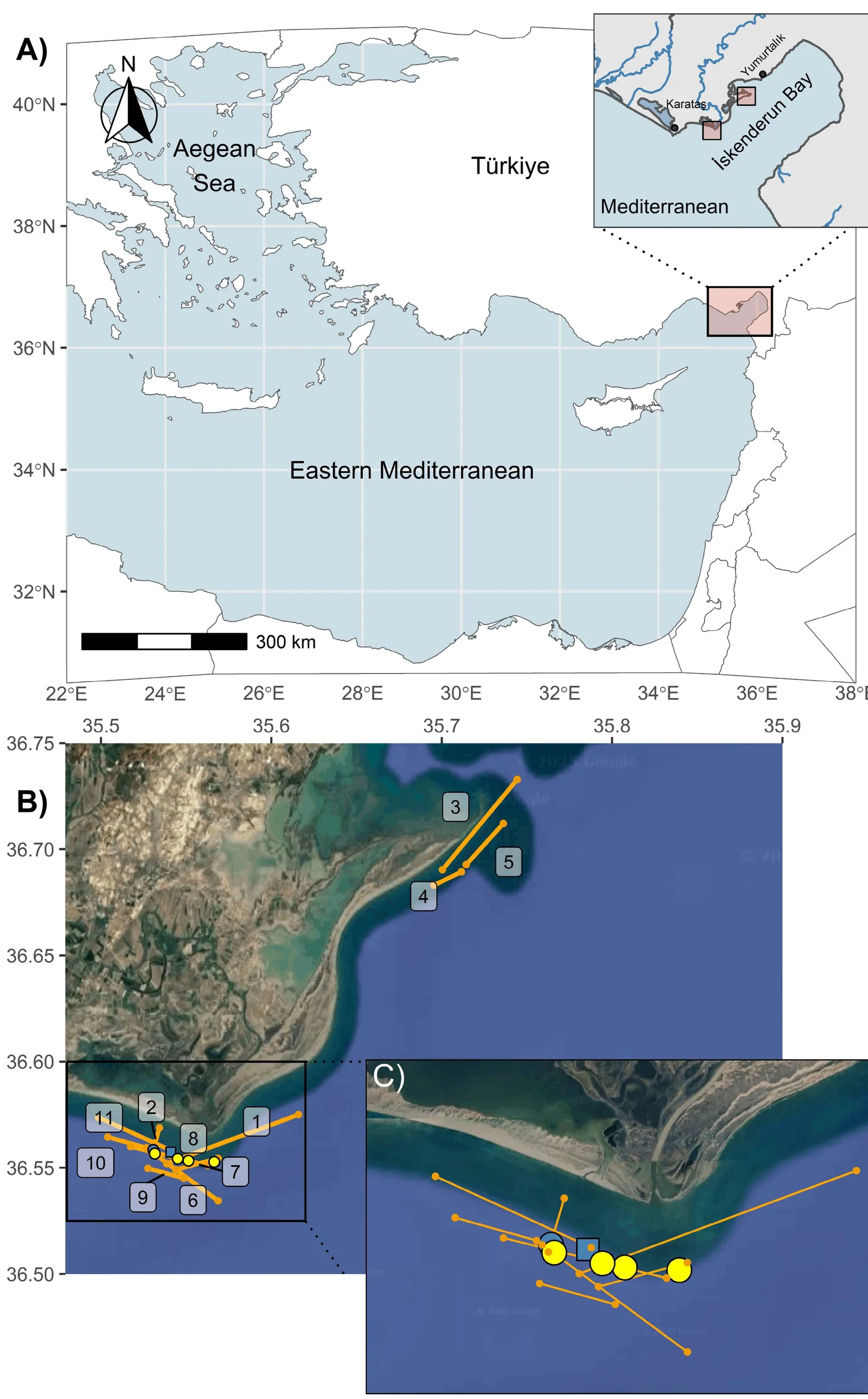
Study area in İskenderun Bay, northeastern Mediterranean Sea (A). Locations of the seven longline deployments conducted during the June 2024 field campaign, with numbers indicating the deployment sequence (B). Enlarged view of the capture and tagging area in Yumurtalık Cove showing the capture locations of the six juvenile *Carcharhinus plumbeus* individuals (blue circles: males; yellow circles: females). The rectangle indicates the individual equipped with the MiniPAT satellite tag (C).

The surveyed areas included Yumurtalık Cove and the western coasts of Iskenderun Bay (Fig. 1A). This region represents the most recently identified nursery area for sandbar sharks in the Mediterranean Sea, following Boncuk Cove in Gökova Bay, southwestern Türkiye (Filiz, 2018), and the Gulf of Gabès in southern Tunisia (Bradaï et al., 2005). The study area is characterized by shallow coastal habitats with predominantly sandy and muddy substrates, influenced by the Seyhan and Ceyhan river deltas and associated estuarine environments, which contribute to high biological productivity. Surveyed habitats generally ranged from 5 to 20 m depth and include coastal waters adjacent to lagoon systems and river mouths. The area is also influenced by multiple human activities, including commercial fisheries, marine traffic, and coastal development, which may exert localized environmental pressure on these coastal ecosystems (Yilmaz & Sönmez, 2018).

### Fishing operations

A 13-m commercial fishing vessel was used during the field campaign conducted between 5 and 12 June 2024. The vessel is routinely used for commercial longline and set-net fisheries and operated with a crew of three to four professional fishers. A total of seven fishing trips were conducted from the harbours of Karataş and Yumurtalık (Fig. 1B). Under standard commercial practice, the vessel’s longline system is configured for grouper fisheries. For the present study, the gear was modified into a semi-demersal longline by incorporating floats along the mainline to achieve partial lift from the seabed. Additional fishing configurations were also evaluated, including the deployment of a custom heavy-duty longline equipped with a strengthened mainline and larger hooks. The traditional semi-demersal longline consisted of a 210d multi 140 monofilament polyamide mainline equipped with 800 Mustad 7/0 hooks (19 × 20 × 43 mm), whereas the custom longline was equipped with 200 VMC 3/0 hooks. Additional floats were attached at regular intervals along the mainline to maintain the desired fishing depth above the seabed. Setting and hauling operations were performed by professional fishers. Fishing operations lasted approximately 7–8 h per day at depths ranging from 6 to 20 m. Sardine and mullet were used as bait on all hooks. A detailed summary of the longline configurations is provided in Table S1.

### Handling and data collection onboard

All captured elasmobranchs were brought aboard over the stern using a hoop net. The hook was carefully and swiftly removed from their mouths to minimize injury. For sharks, a water hose was inserted into the mouth to aid oxygenation, a towel was placed over the eyes to reduce stress, and all necessary precautions were taken to prevent bites. Each individual was visually sexed based on sexual dimorphism, and various morphometric measurements were recorded using a measuring tape: precaudal length (PCL), fork length (FL), and stretch total length (sTL). Other elasmobranch species were sexed, measured, and released under a minute.

### Satellite tagging

For suitably sized sharks (>one m total length and in good condition), a MiniPAT pop-up satellite archival tag (Wildlife Computers, Redmond, WA, USA) was externally attached to the dorsal musculature near the base of the first dorsal fin using the manufacturer’s recommended attachment procedure. MiniPAT tags archive high-resolution depth, temperature, and light data internally, transmit summarized time-series *via* Argos after pop-up, and use light-level and sea surface temperature (SST) data for daily geolocation estimates. The MiniPAT tag was programmed to record depth and temperature time-series data at a sampling interval of 10 min (600 s). A corrodible burn pin releases the tag from the tether on a pre-programmed date (here, 90 days) or when the MiniPAT detects it is no longer attached to the animal. Once released, the tag floats on the ocean surface and transmits summarized data *via* Argos satellites. Active premature release was activated (in case of tag floating at the surface or at constant depth for longer than 3 days or if it is at a depth below 1,700 m). The MiniPAT also recorded the daily proportion of time spent within predefined temperature and depth bins throughout the tracking period. Following tag pop-up and data transmission, the processed datasets generated by the Wildlife Computers Data Portal contained synchronized geolocation estimates and their corresponding timestamps (yearday/date-time), together with depth and temperature records. Daily geolocation estimates were generated using the GPE3 algorithm (Wildlife Computers), which integrates twilight, SST, and dive depth observations with corresponding environmental reference data to generate daily gridded probability surfaces. The most likely latitude and longitude were then extracted from these probability surfaces for subsequent analyses (Hammerschlag, Gallagher & Lazarre, 2011).

### Data analysis

All analyses were completed using the R statistical environment (R Core Team, 2021). Morphological data and capture depth were compared between males and females. As GPE3 positions are modelled geolocation estimates derived from light-level data and not Argos satellite fixes, no Argos-specific quality classes or filtering procedures were applied. Given the study is based on a single individual with relatively low temporal resolution (one Argos-derived location every 6 h in average), fine-scale movement metrics (*e.g.*, movement rates or residence time) were not considered robust and were therefore not computed; the analysis remains descriptive and focuses on overall movement trajectories and displacement patterns. The farthest distance was calculated as the maximum straight-line (Euclidean) distance between the shark’s tagging location (used as the fixed starting point) and any subsequent recorded position during the tracking period. Temperature and depth ranges recorded by the MiniPAT sensors were analyzed by examining the time spent in each temperature and depth bin using boxplots showing the median, interquartile range, and variability among daily values.

The animal study protocol was approved by Committee of Health Sciences Experimental Application and Research Center of Çukurova University (ÇUSUBIDAM) (protocol code 2/9-15.02.2024).

A research authorization (E-67852565-140.03.03-13185281) was granted by the Ministry of Foreign Affairs and the General Directorate of Fisheries and Aquaculture, Ministry of Agriculture and Forestry of the Turkish Republic, along with approval for the participation of foreign researchers (E-67852565-140.03.03-13949260).

An experimental fishing permit was granted to us by Ministry of Foreign Affairs (21.03.2024 date and E-21252218-300.04.37765712) and the General Directorate of Fisheries and Aquaculture, Ministry of Agriculture and Foresty of the Turkish Republic to collect elasmobranchs in the Yumurtalık locations during the June (18/04/2024-E.983970).

## Results

### Onboard captures

The total effort (involving retrieving the longline) was a cumulative duration of 23 h and 15 min over 7 different days (19 h and 15 min with the traditional longline + 4 h with custom longline). A total of six juvenile sandbar sharks (two males and four females) were captured at depths of 7 to 17 m, with stretch total lengths ranging from 66 to 120 cm (Table 1, Fig. 1B).

**Table 1:** Detailed information on the individuals of sandbar shark (*C. plumbeus*) captured during the field campaign. Morphometric measurements, sex, capture depth, and tagging status of the six juvenile *Carcharhinus plumbeus* individuals captured during the June 2024 field campaign in Yumurtalık Cove, eastern Mediterranean Sea.

Capture date	Capture time	Release time	Location	Depth (m)	Sex	Pre caudal length (PCL) (cm)	Fork length (FL) (cm)	stretch total length (sTL) (cm)	Satellite tag deployment
11/06/2024	09:35	09:37	36.558°N, 35.531°E	9	M	72.5	76	89	/
11/06/2024	09:49	09:53	36.557°N, 35.541°E	8	M	74	84.5	105	MiniPAT (23P0161)
11/06/2024	12:10	12:17	36.553°N, 35.567°E	17	F	92	98	120	/
12/06/2024	09:50	09:52	36.553°N, 35.551°E	8	F	-	-	96	/
12/06/2024	10:11	10:13	36.554°N, 35.545°E	7	F	69	76.5	86.5	/
12/06/2024	10:56	10:58	36.557°N, 35.532°E	7	F	47.5	54	66	/

### Satellite tracking

**Figure 2:**
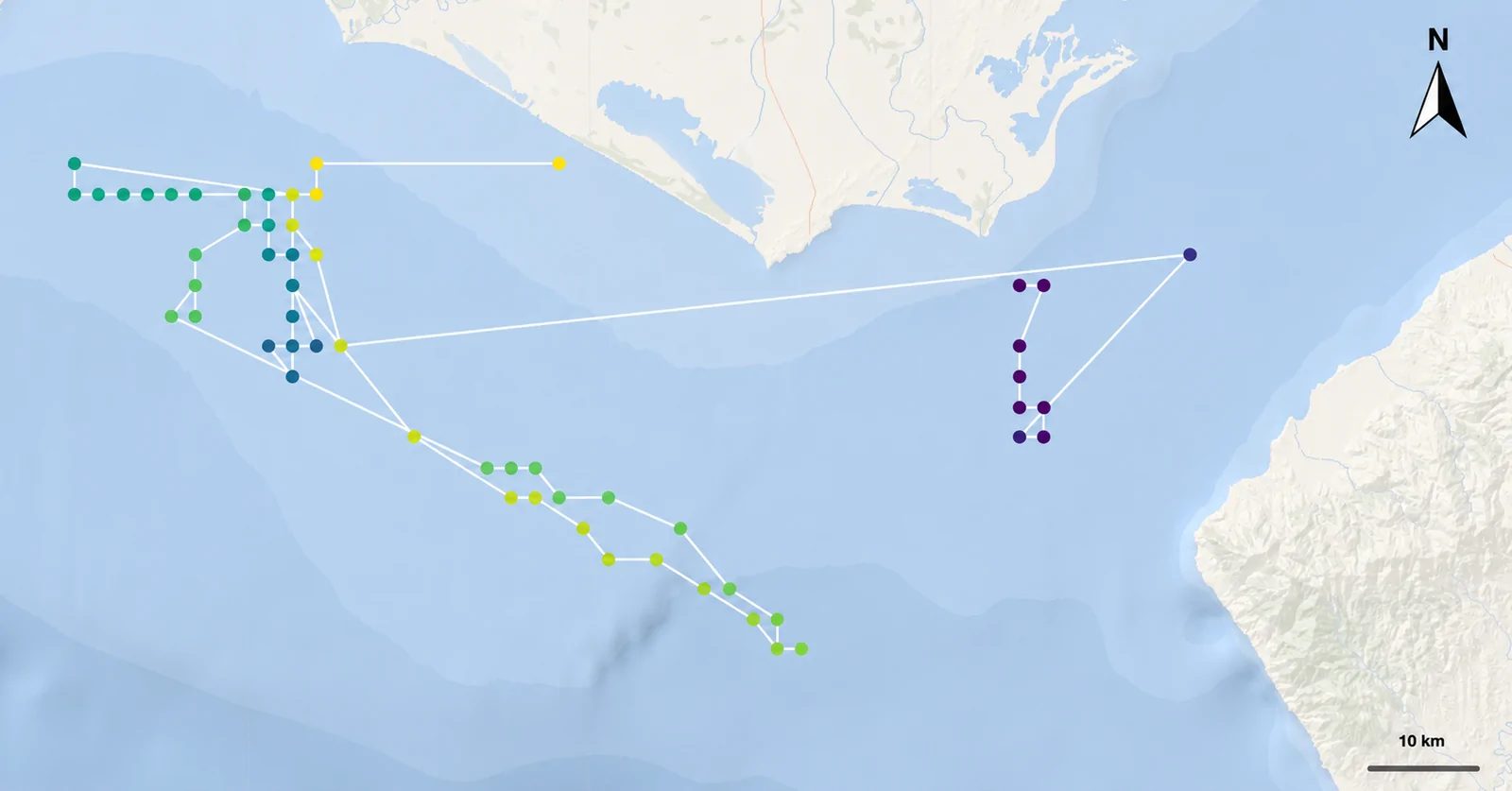
Trajectory of the sandbar shark (*C. plumbeus*) recorded between June 11 and July 31, 2024. Reconstructed movement trajectory of the juvenile sandbar shark (*Carcharhinus plumbeus*) equipped with a MiniPAT satellite tag in Yumurtalık Cove, eastern Mediterranean Sea. Daily geolocation estimates generated by the GPE3 algorithm are shown for the 51-day tracking period (11 June–31 July 2024). The colour gradient represents time, with darker colours indicating the beginning of the trajectory and lighter colours showing the most recent locations.

**Figure 3:**
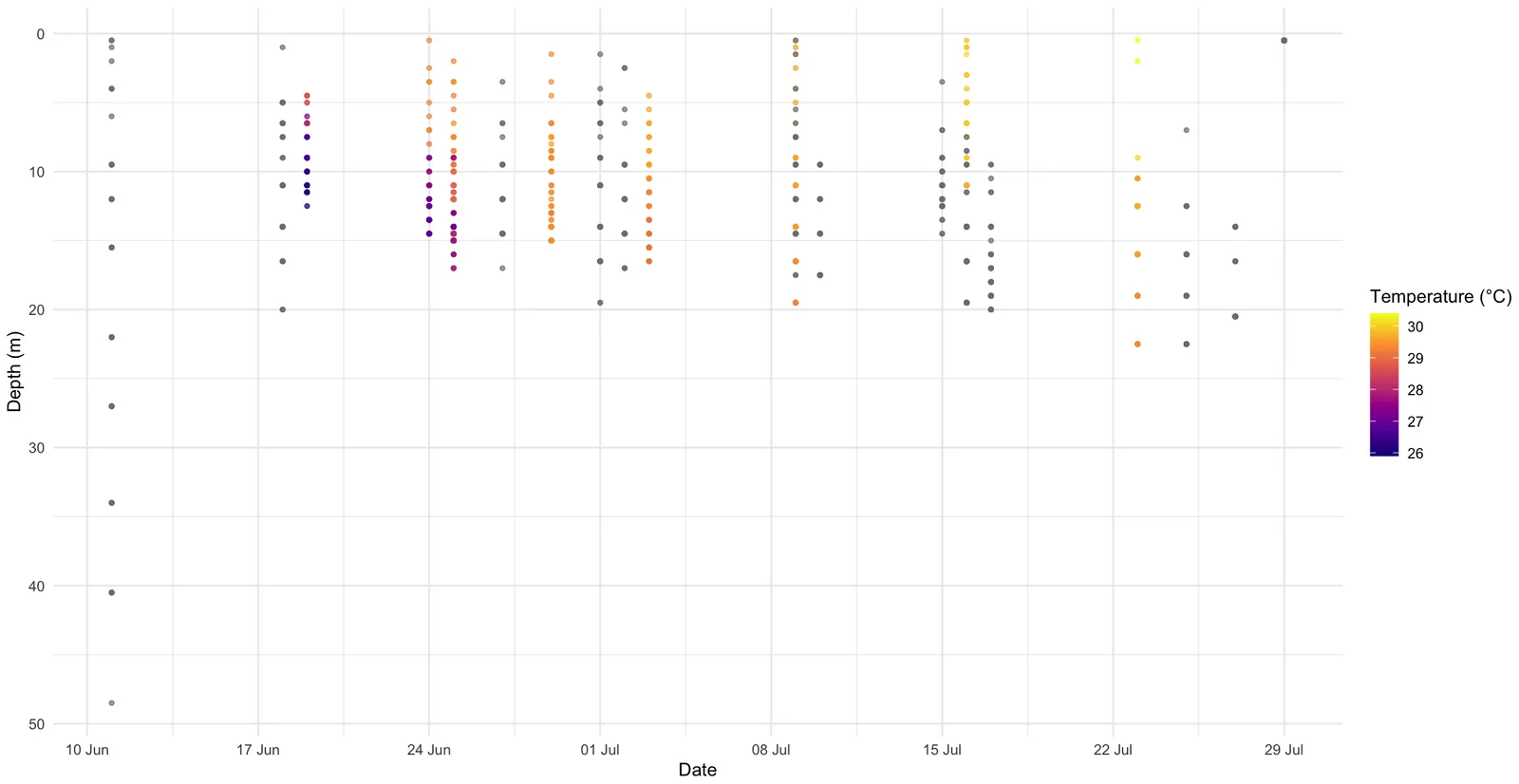
Temporal variation in depth and associated temperatures recorded by the logger attached to the sandbar shark (*C. plumbeus*). Grey dots indicate unavailable temperature data. Temporal variation in depth and associated water temperature recorded by the MiniPAT tag attached to the juvenile sandbar shark (*Carcharhinus plumbeus*) during the 51-day tracking period. Grey dots indicate depth records for which corresponding temperature measurements were unavailable.

**Figure 4:**
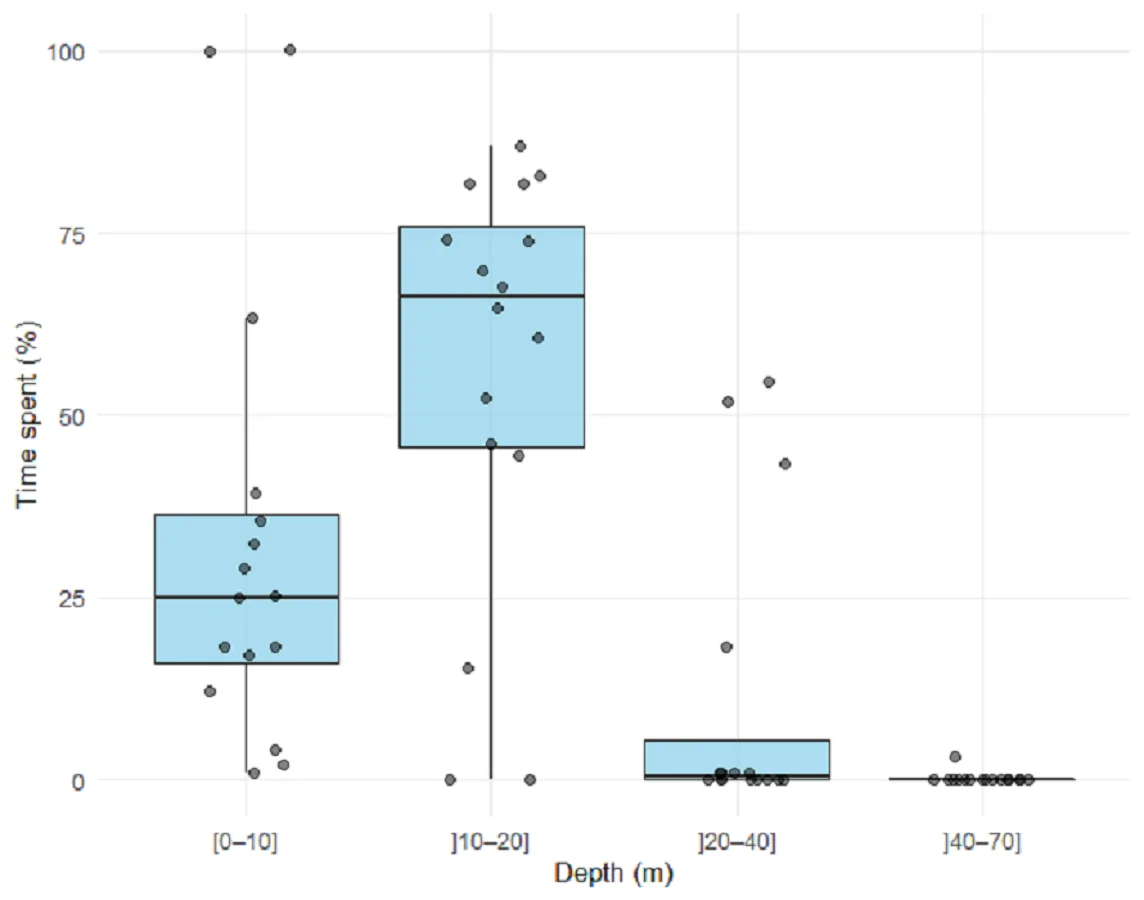
Distribution of the daily proportion of time spent per depth range by the tagged sandbar shark (*C.plumbeus*). Distribution of the daily proportion of time spent within predefined depth intervals by the tagged juvenile sandbar shark (*Carcharhinus plumbeus*) throughout the tracking period. Boxplots show the median, interquartile range, and overall variability of the daily proportion of time spent within each depth class.

A total of six juvenile sandbar sharks (two males and four females) were captured during the present field campaign, and the morphometric characteristics of all captured individuals are summarized in Table 1. One individual, 105 cm sTL male, met the minimum size requirement for tagging and was equipped with a MiniPAT. Handling, data collection, tagging, and release were completed efficiently in less than 7 min. Although one additional individual exceeded the minimum size criterion for tagging, it exhibited signs of poor post-capture condition, including reduced responsiveness, and was therefore released immediately after measurements to minimize additional handling stress. The sandbar shark individual was tagged in the coastal waters near Yumurtalık (Fig. 2). The tagged sandbar shark was tracked for 51 days, from 11 June to 31 July 2024, using the processed geolocation estimates generated by the Wildlife Computers GPE3 algorithm. The reconstructed trajectory indicated that the individual initially moved offshore before travelling southwest, exhibiting a combination of localized movements and more directed displacements. The individual then traveled southwest, covering a broader area with a combination of directed movements and localized activity. Eventually, the individual returned closer to the coast. The maximum straight-line distance from the tagging site to the farthest recorded location was approximately 88 km. On the first day of tracking (11 June 2026), the logger recorded depth data ranging from 0.5 m to 48.5 m (the maximum depth reached during the tracking) without corresponding temperature measurements (Fig. 3). From the following day until the end of the tracking period (29 July), the data show that the individual dove to a maximum depth of 22.5 m, moving within a temperature range of 25.9 °C to 30.4 °C (Fig. 3). Additionally, the daily time spent per depth range recorded by the logger indicates that the individual spent most of its time between 10 and 20 m depth, with median values around 65% of the time. Considerable variability was observed in the shallowest range (0–10 m), suggesting occasional use of surface waters, while time spent below 20 m was negligible (Fig. 4).

## Discussion

Yumurtalık, already established as a breeding ground for sandbar sharks (Başusta, Başusta & Ozyurt, 2021), was further confirmed by the capture of six young individuals during the present field campaign. The successful deployment of one MiniPAT satellite tag has provided valuable in-sights into the horizontal movements of the sandbar shark, yet the premature detachment of a tag underscores a significant limitation in telemetry studies (Conrath & Musick, 2010). The movement patterns observed in the tracked juvenile reveal a combination of coastal and offshore activities, depth and temperature preference, a behavior that aligns with findings in other studies on sandbar sharks and other shark species, where site fidelity and localized movement are common among juveniles (Rechisky & Wetherbee, 2003; Oh et al., 2017; Ferretti et al., 2024). This behavior may be associated with prey availability, habitat suitability, and predator avoidance, as noted in studies on other juvenile shark populations (Conrath & Musick, 2010; Speed et al., 2010). The observed range of approximately 88 km from the tagging location to the farthest recorded point suggests a relatively small home range for the individual, indicating a semi-resident or resident pattern rather than a migratory one (Oh et al., 2017). This is consistent with previous findings that juvenile sandbar sharks tend to remain within nursery areas, likely due to tidal movements, optimal foraging conditions, and lower predation risk (Conrath & Musick, 2010; Oh et al., 2017; Ferretti et al., 2024). Similar patterns of residency have been documented in juvenile sandbar sharks inhabiting nursery grounds in Delaware Bay and Chesapeake Bay, where individuals repeatedly used restricted coastal areas over periods of weeks to months (Rechisky & Wetherbee, 2003; Conrath & Musick, 2010). These findings suggest that nursery habitats provide favourable environmental conditions that support juvenile survival and development (Heupel, Carlson & Simpfendorfer, 2007). The restricted movements observed in the present study are therefore consistent with the expected behaviour of juvenile sandbar sharks and further support the ecological importance of Yumurtalık Cove as a nursery habitat in the eastern Mediterranean.

The depth recorded by the MiniPAT deployed on one individual indicate a clear preference for shallow coastal waters between 10 and 20 m depth. This result is consistent with the capture depths of the six individuals caught and released at sea, which ranged between 7 and 18 m (Table 1). The tagged shark also spent periods near the surface and only rarely reached depths of 20 m, suggesting occasional movements within the upper water column and limited excursions into slightly deeper waters. The occurrence of sharks in shallow coastal waters (0–20 m depth) may be explained by several non-mutually exclusive hypotheses, including coastal habitat use, nursery behaviour, exploitation of feeding opportunities in shallow habitats, and predator avoidance (Speed et al., 2010).

**Figure 5:**
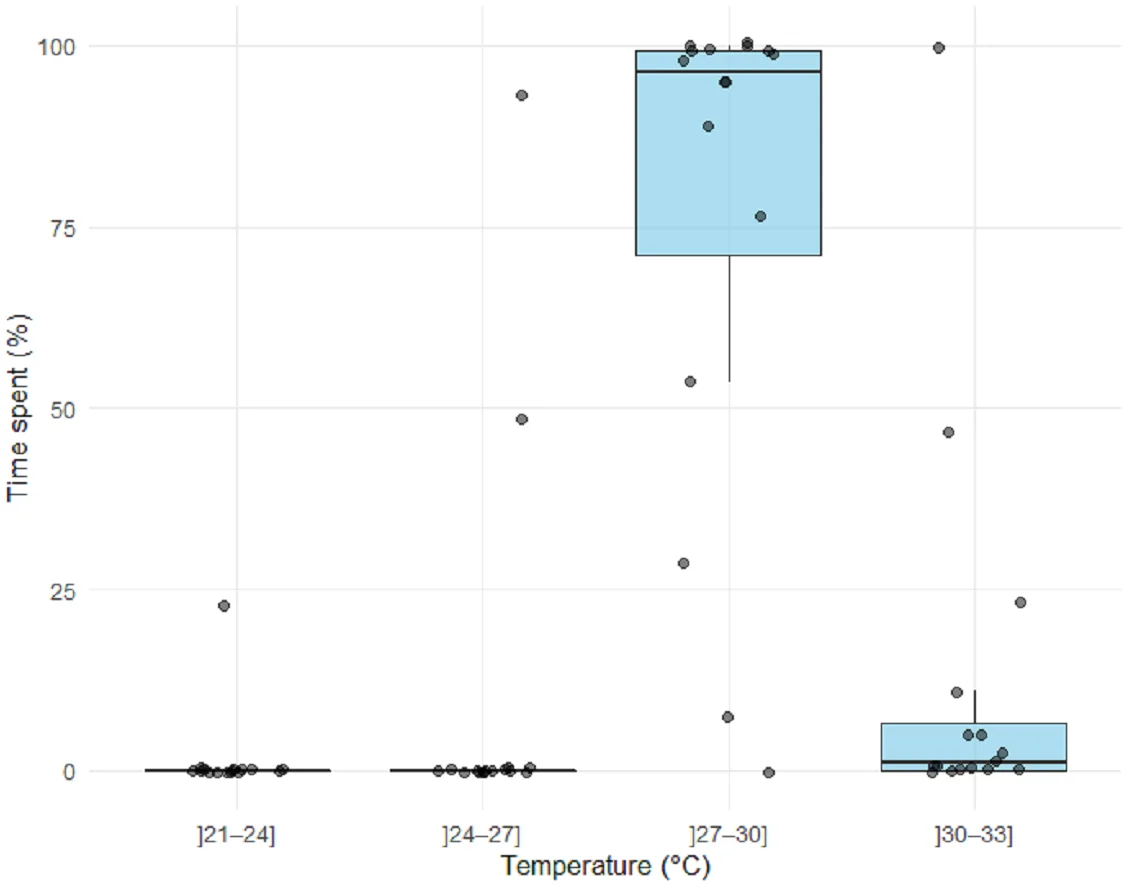
Distribution of the daily proportion of time spent per temperature range by the tagged sandbar shark (*C. plumbeus*). Distribution of the daily proportion of time spent within predefined water temperature intervals by the tagged juvenile sandbar shark (*Carcharhinus plumbeus*) throughout the tracking period. Boxplots show the median, interquartile range, and overall variability of the daily proportion of time spent within each temperature class.

Regarding the daily time spent across temperature ranges, the tagged juvenile spent the majority of the tracking period in waters between 27 °C and 30 °C, while only a small proportion of time was recorded in waters cooler than 27 °C or warmer than 30 °C (Fig. 5). Although this pattern may reflect the prevailing summer thermal conditions of the study area, it should not be interpreted as evidence of species-level thermal preference because it is based on a single individual tracked over a limited period. Similar observations should therefore be considered preliminary until supported by additional telemetry studies involving larger sample sizes and multiple seasons.

Key limitations of this study include the small sample size, as a single individual cannot represent the overall behaviour of the local population, and the short tracking duration, which does not exclude the possibility of broader movements beyond the Gulf of Iskenderun. The first major challenge was locating sharks at sea, a task that can remain difficult even in areas where the species is known to occur. In this case, juvenile sandbar sharks had been observed in the area during July and August according to local fishers’ information that shared also photographs of neonates. Based on this information, the mission was scheduled for early June, with the hope of encountering adult females that had recently given birth and might still be present in the area. However, only small juveniles were captured, which limited the number of deployed tags, as we had decided to tag only individuals larger than one meter to minimize potential invasiveness of the method. Similar fieldwork constraints have been reported in other tagging studies where the rarity or elusive behavior of sharks made encounters unpredictable (Dicken, Booth & Smale, 2006). Another limitation concerns the tracking duration, which in this study lasted only about six weeks due to premature tag detachment, although the tag had been programmed for a 90-day deployment. Potential causes of early release include biofouling, tag shedding, attachment failure, or mechanical damage (Vázquez-Aguilar et al., 2025). An additional limitation is the positional uncertainty inherent to light-based PSAT geolocation estimates. Consequently, the reconstructed trajectory should be interpreted as an approximation of the shark’s movement at the regional scale rather than an exact representation of its position throughout the deployment. However, despite this inherent uncertainty, the estimated locations consistently support the interpretation that the tagged individual remained within the eastern Mediterranean coastal system during the tracking period, and therefore the principal conclusion of restricted coastal movements is unlikely to be affected. Given these limitations, and considering the high cost of satellite telemetry equipment, this type of study remains logistically and financially challenging (Vázquez-Aguilar et al., 2025), and has so far been rarely applied to elasmobranchs in the Mediterranean Sea. Nevertheless, satellite telemetry provides valuable insights into the ecology of elusive species that are otherwise difficult to study in their natural habitat, though it remains insufficient on its own to fully characterize habitat use for conservation purposes. When direct access to animals in the field is limited, complementary approaches should be used to provide additional information that improves our understanding of species distribution and behavior, such as incorporating local ecological knowledge from fishers (Barbato et al., 2021) or citizen science (Gotama et al., 2023). Once shark aggregations are identified through these methods, satellite tagging can then be applied, reducing the risk of unsuccessful search efforts at sea. Yumurtalık was selected for this study precisely because it is recognized as a nursery area. The remaining tags will be deployed during upcoming missions in other Mediterranean regions, focusing on sites where sharks are known to occur.

## Conclusions

The discovery of nursery grounds in Yumurtalık Cove reinforces the growing recognition of key habitats essential to the survival of sandbar sharks in the Mediterranean, joining other confirmed sites such as the Gulf of Gabès and Boncuk Cove. Protecting these areas from fisheries pressure and habitat degradation is essential for the conservation of this endangered species. Given the high bycatch rates in coastal fisheries and the particular vulnerability of juvenile sharks, implementing spatial management measures could mitigate the impact on these essential habitats.

Future studies involving multiple tagged individuals tracked over extended periods are essential to gain a more comprehensive understanding of habitat use and connectivity between nursery areas and other coastal zones in the Mediterranean. Migratory species often cross national borders and require international cooperation and coordinated conservation measures. Collaborative initiatives will be key to advancing effective conservation strategies for sandbar sharks and other elasmobranchs across the region. Future telemetry studies should include larger sample sizes, multiple life stages, and longer tracking periods to better characterize movement variability and habitat connectivity across the Mediterranean. Integrating telemetry with complementary information, including habitat mapping, environmental monitoring, and nursery habitat assessments, will further improve our understanding of the ecological requirements of this endangered species and support evidence-based conservation planning (Özgül et al., 2024; Barbato et al., 2023).

## Supplemental Information

10.7717/peerj.21658/supp-1 Supplemental Information 1 ARRIVE 2.0 checklist

10.7717/peerj.21658/supp-2 Supplemental Information 2 Dates, departure ports and longline specifications used each fisheries operation

## Additional Information and Declarations

### Field Study Permissions

The following information was supplied relating to field study approvals (i.e., approving body and any reference numbers): A research authorization for the field experiments (E-67852565-140.03.03-13185281) was granted by the Ministry of Foreign Affairs and the General Directorate of Fisheries and Aquaculture, Ministry of Agriculture and Forestry of the Turkish Republic, along with approval for the participation of foreign researchers (E-67852565-140.03.03-13949260). An experimental fishing permit was granted to us by Ministry of Foreign Affairs (21.03.2024 date and E-21252218-300.04.37765712) and the General Directorate of Fisheries and Aquaculture, Ministry of Agriculture and Foresty of the Turkish Republic to collect elasmobranchs in the Yumurtalık locations during the June (18/04/2024-E.983970)

### Data Availability

The following information was supplied regarding data availability: The data is available at Zenodo: Başusta, N., Mendez, L., Soldo, A., Ozyurt, C. E., Mavruk, S., Ozgul, A., Lok, A., de Sabata, E., Clò, S., Bengil, E. G.& Jacquesson, E. (2026). First Satellite Tracking of a Juvenile Sandbar Shark (*Carcharhinus plumbeus*) off the Mediterranean Coast of Türkiye [Dataset]. Zenodo. https://doi.org/10.5281/zenodo.18619094
