# Supplementary material for: First Satellite Tracking of a Juvenile Sandbar Shark (Carcharhinus plumbeus) off the Mediterranean Coast of Türkiye
Source: PeerJ. 2026 Aug 18;14:e21658. doi: 10.7717/peerj.21658 (PMC13499531; doi:10.7717/peerj.21658)
Supplement: Supplementary file 2 [file peerj-14-21658-s002.pdf]

Table S1 Dates, departure ports and longline specifications used each fisheries operation.

Date	Port of departure	Longline specifications
05/06/24	Karataş	Traditional longline: 1 mm monofilament line (polyamide) / 9 km length / 800 hooks (size: 19 x 20 x 43 mm / Mustad 7/0) / 4 buoys every 400 m (100 hooks) + 4 weights / 0.9 mm monofilament line branches spaced of 6 m / line branches: 1.2 m
06/06/24	Karataş	Traditional longline: 1 mm monofilament line (polyamide) / 7 km length / 650 hooks (size: 19 x 20 x 43 mm / Mustad 7/0) / 4 buoys every 400 m (100 hooks) + 4 weights / 0.9 mm monofilament line branches spaced of 6 m / line branches length: 1.2 m
08/06/24	Yumurtalik	Traditional longline: 1 mm monofilament line (polyamide) / less than 7 km length / 500 hooks (size: 19 x 20 x 43 mm / Mustad 7/0) / 4 buoys every 400 m (100 hooks) + 4 weights / 0.9 mm monofilament line branches spaced of 6 m / line branches length: 1.2 m
10/06/24	Yumurtalik	Traditional longline: 1 mm monofilament line (polyamide) / less than 7 km length / 500 hooks (size: 19 x 20 x 43 mm / Mustad 7/0) / 4 buoys every 400 m (100 hooks) + 4 weights / 0.9 mm monofilament line branches spaced of 6 m / line branches length: 1,2 m<br>Custom longline: monofilament polyamide 210d multi 140 / 200 hooks (size: VMC 3/0) / 1 buoy every 40-50 hooks / line branches length: 4 m (3 m monofilament + 1 m steel wire)
11/06/24	Karataş	Traditional longline: 1 mm monofilament line (polyamide) / less than 7 km length / 500 hooks (size: 19 x 20 x 43 mm / Mustad 7/0) / 4 buoys every 400 m (100 hooks) + 4 weights / 0.9 mm monofilament line branches spaced of 6 m / line branches length: 1.2 m<br>Custom longline: monofilament polyamide 210d multi 140 / 200 hooks (size: VMC 3/0) / 1 buoy every 10 hooks / line branches length: 4 m (3 m monofilament + 1 m steel wire)
12/06/24	Karataş	Traditional longline: 1 mm monofilament line (polyamide) / less than 7 km length / 500 hooks (size: 19 x 20 x 43 mm / Mustad 7/0) / 4 buoys every 400 m (100 hooks) + 4 weights / 0.9 mm monofilament line branches spaced of 6 m / line branches length: 1.2 m<br>Custom longline: monofilament polyamide 210d multi 140 / 200 hooks (size: VMC 3/0) / 1 buoy every 10 hooks / line branches length: 4 m (3 m monofilament + 1 m steel wire)
13/06/24	Karataş	Traditional longline: 1 mm monofilament line (polyamide) / less than 7 km length / 500 hooks (size: 19 x 20 x 43 mm / Mustad 7/0) / 4 buoys every 400 m (100 hooks) + 4 weights / 0.9 mm monofilament line branches spaced of 6 m / line branches length: 1.2 m<br>Custom longline: monofilament polyamide 210d multi 140 / 200 hooks (size: VMC 3/0) / 1 buoy every 10 hooks / line branches length: 4 m (3 m monofilament + 1 m steel wire)
